# β-cyclocitral as an apocarotenoid stress signal: biosynthesis, hormonal crosstalk, and emerging omics perspectives in plant abiotic stress tolerance

**DOI:** 10.3389/fpls.2026.1903199

**Published:** 2026-08-05

**Authors:** Pravej Alam, Mohammad Faizan, Thamer Albalawi, Shamweel Ahmad, Altafur Rahman, Yawar Habib

**Affiliations:** 1 Department of Biology, College of Science and Humanities, Prince Sattam bin Abdulaziz University, Alkharj, Saudi Arabia; 2 Botany Section, School of Sciences, Maulana Azad National Urdu University, Hyderabad, India; 3 Center of Plant Systems Biology and Biotechnology, Plovdiv, Bulgaria; 4 Center for Bio and Medical Technologies, Skolkovo Institute of Science and Technology, Moscow, Russia

**Keywords:** oxidative stress, phytohormone interaction, plant-adaptive mechanism, ROS homeostasis, stress-responsive signaling, β-cyclocitral

## Abstract

Abiotic stress, including drought, salinity, heavy metals, and extreme temperatures, severely limits plant growth, productivity, and survival. These stresses frequently occur simultaneously and disrupt cellular homeostasis, photosynthesis, and metabolic processes. To cope with such adverse conditions, plants activate complex physiological, biochemical, and molecular defense mechanisms. Among the emerging stress-related signaling compounds, β-cyclocitral, a β-carotene-derived apocarotenoid, has gained significant attention due to its crucial role in plant stress adaptation. β-Cyclocitral enhances photoprotection by scavenging free radicals and reducing singlet oxygen-mediated damage to photosynthetic machinery. It also modulates reactive oxygen species (ROS) homeostasis through the activation of antioxidant defense systems, thereby minimizing oxidative stress. In parallel, key phytohormones such as abscisic acid (ABA), jasmonic acid (JA), and salicylic acid (SA) regulate diverse stress-responsive pathways that improve plant tolerance and defense. Increasing evidence suggests that β-cyclocitral interacts closely with these hormonal signaling networks to coordinate stress responses and metabolic adjustments. This review highlights recent advances in the biosynthesis, physiological functions, and signaling roles of β-cyclocitral, with particular emphasis on its mechanistic crosstalk with ABA, JA, and SA pathways in enhancing abiotic stress tolerance in plants.

## 1 Introduction

The world’s food supply is in jeopardy due to abiotic stress, which has decreased agricultural output and cultivated land. Horticultural crops are negatively impacted by abiotic stress challenges, including waterlogging, salinity, extreme temperatures, drought, and heavy metals (Yin et al., 2023; Faizan et al., 2023). These stresses cause oxidative stress, which modifies several physiological processes by inducing the formation of reactive oxygen species (ROS) (Alam et al., 2025). Carotenoids are a diverse group of pigmented isoprenoids, comprising over 600 compounds (Britton, 2022). Although these compounds are distributed across various biological divisions, they are synthesized *de novo* only by photosynthetic organisms (plants and algae) and by certain fungi and bacteria, whereas animals cannot synthesize carotenoids and must obtain them from the diet. Carotenoids are essential components of the photosynthetic apparatus in plants, functioning as photoprotective pigments and contributing to light harvesting during photosynthesis. Furthermore, the breakdown products of β-carotene act as bioactive compounds that perform important physiological functions in plants. Carotenoids are also responsible for the vibrant orange, reddish-brown, and yellow coloration observed in many flowering and fruiting plants. In addition to enzymatic cleavage, apocarotenoids can also be generated non-enzymatically through ROS-mediated oxidation, which contributes to diverse biological functions (Imtiaz et al., 2023).

One carotenoid-derived byproduct that may arise under either of these circumstances is β-cyclocitral, which is generated through the oxidation of β-carotene by singlet oxygen (¹O_2_) (D'Alessandro et al., 2018). To date, two major functions of carotenoids have been recognized: enhancing light absorption and protecting photosynthetic components from ROS (Triantaphylidès and Havaux, 2009). Beyond these two roles, carotenoid oxidation products act as signals in their own right: exogenous β-cyclocitral reprograms the expression of a large set of singlet-oxygen-responsive genes (SORGs) in *Arabidopsis*. Mechanistically, β-cyclocitral acts as a secondary messenger that relays the ¹O_2_ signal generated in the chloroplast to the nucleus; part of this transduction requires the small nuclear zinc-finger protein METHYLENE BLUE SENSITIVITY 1 (MBS1), which functions downstream of β-cyclocitral and is required for the transcriptional reprogramming of ¹O_2_-responsive genes (Shumbe et al., 2017; Dogra et al., 2018).

β-Cyclocitral has been detected in many plant species, including rice, grapes, parsley, tea, and several trees. β-Cyclocitral is also a fragrance compound with a distinctive, hay-like, moderately fragrant aroma (Radwan et al., 2025). According to recent investigations, plants are protected against drought by the exogenous application of β-cyclocitral. Collectively, these findings indicate that β-cyclocitral can trigger numerous stress signals. β-Cyclocitral acts as an oxidative product that regulates nuclear gene expression under excess-light stress, alongside carotenoid oxidation. Under salt stress conditions, β-cyclocitral was also shown to have a beneficial effect by preventing the detrimental impact of salt on rooting depth and by positively affecting plant vigor (Carnà et al., 2025).

This review highlights the pivotal functions of β-cyclocitral in enhancing plant resistance to a wide range of abiotic stresses, with particular emphasis on the intricate crosstalk between β-cyclocitral and the key phytohormones abscisic acid (ABA), jasmonic acid (JA), and salicylic acid (SA). By examining how β-cyclocitral integrates with these hormonal signaling pathways, the review provides deeper insight into the coordinated regulatory networks that strengthen stress tolerance in crop plants. Furthermore, it underscores recent advances in β-cyclocitral biosynthesis, signaling dynamics, and its potential applications in developing climate-resilient agricultural systems. Recent reviews have surveyed apocarotenoid biosynthesis and the stress-signalling roles of β-cyclocitral (Carnà et al., 2025; Lachica et al., 2026). Building on these, the present review is set apart by its focus on how β-cyclocitral is integrated with the ABA, SA and JA signalling networks, read together with emerging transcriptomic, proteomic and metabolomic evidence, and by extending the discussion to roles that have so far received less attention, including nodulation, root organogenesis, senescence and fruit ripening. Throughout, we have tried to make clear which of these links are supported by direct experimental evidence and which remain working hypotheses.

## 2 Biosynthesis of β-cyclocitral

**Figure 1:**
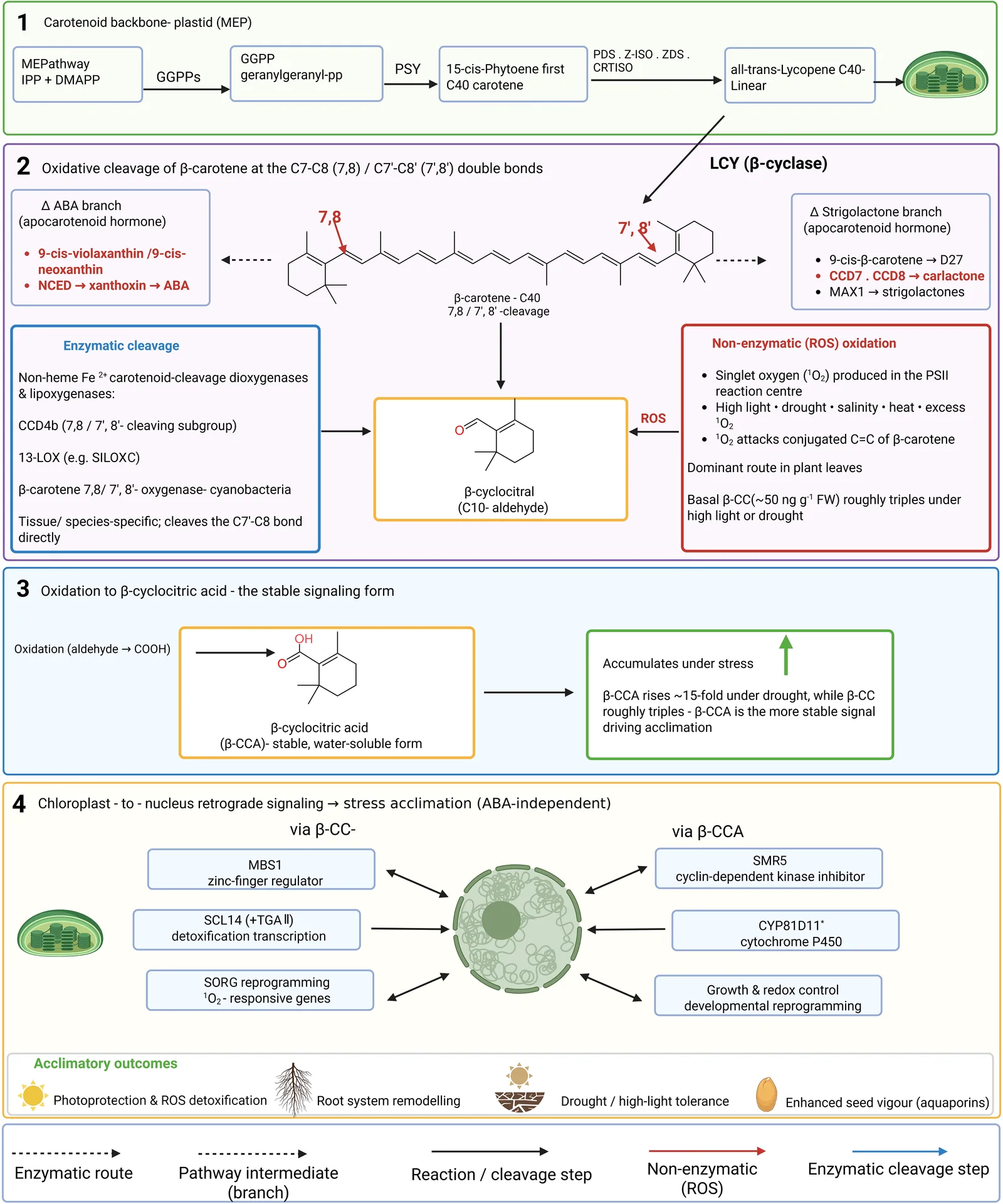
Biosynthetic pathway of β-cyclocitral and β-cyclocitric acid (β-CCA) from β-carotene. The four panels show (1) plastidial synthesis of β-carotene via the MEP pathway; (2) cleavage of β-carotene at the 7, 8/7′, 8′ bonds by CCDs/lipoxygenases or by PSII-derived singlet oxygen (¹O_2_), with the parallel ABA and strigolactone branches; (3) oxidation of β-cyclocitral to the stable, mobile β-CCA; and (4) chloroplast-to-nucleus retrograde signalling driving photoprotection and stress acclimation. Arrow conventions are given in the key at the foot of the figure.

β-Cyclocitral originates from the carotenoid biosynthetic pathway and is released from β-carotene by oxidative cleavage. In the plastid, the methylerythritol phosphate (MEP) pathway supplies isopentenyl diphosphate (IPP) and dimethylallyl diphosphate (DMAPP), which are condensed by geranylgeranyl diphosphate synthase (GGPPS) to form geranylgeranyl diphosphate (GGPP). Two GGPP molecules are then joined by phytoene synthase (PSY) to produce 15-*cis*-phytoene, the first C40 carotene. Successive desaturation and isomerization by phytoene desaturase (PDS), ζ-carotene isomerase (Z-ISO), ζ-carotene desaturase (ZDS), and carotenoid isomerase (CRTISO) yield all-*trans*-lycopene, which is cyclized by lycopene β-cyclase (LCYB) to β-carotene. β-Carotene is subsequently cleaved at the 7, 8 (7′, 8′) double bonds to release the C10 aldehyde β-cyclocitral. This cleavage proceeds by two routes: an enzymatic route catalyzed by non-heme iron carotenoid cleavage dioxygenases (CCDs) and lipoxygenases, and a non-enzymatic route driven by ¹O_2_ generated in photosystem II (PSII) under high light, drought, salinity, or heat. The volatile β-cyclocitral is then oxidized to the more stable, water-soluble β-cyclocitric acid (β-CCA), which accumulates strongly under stress and serves as a mobile signaling form (**Figure 1**).

The enzymatic pathway of carotenoid oxidation in plants facilitates the formation of β-cyclocitral, catalyzed by the non-heme iron-dependent enzymes carotenoid cleavage dioxygenases (CCDs), producing apocarotenoids. 9-*cis*-epoxycarotenoid cleavage dioxygenases (NCEDs) specifically cleave the 11, 12 double bond of 9-*cis*-epoxycarotenoids in the ABA biosynthetic pathway, although CCDs and NCEDs do not share substrate specificity (Nambara and Marion-Poll, 2005). Four CCDs have been identified in *Arabidopsis*, but which of them is the principal source of β-cyclocitral *in planta* remains unclear. Notably, exogenous β-cyclocitral restores meristematic cell division in the *ccd1 ccd4* double mutant, indicating that CCD-derived β-cyclocitral is functionally important in roots (Dickinson et al., 2019). Accumulation of carotenoids in plants is regulated at the transcriptional level through biosynthetic gene expression (Enfissi et al., 2017). Notably, β-cyclocitral itself feeds back on carotenoid-pathway gene expression.

Gene expression profiling of the carotenoid pathway following β-cyclocitral treatment revealed significant upregulation of *CitZDS*, *CitDXS*, *CitPSY*, *CitGGPPS*, and *CitBCH*, indicating enhanced upstream regulation of carotenoid biosynthesis. Metabolic pathway alterations and changes in intermediate compounds also contributed to modification of carotenoid content. Additionally, downstream upregulation of *CitZEP* promoted suspension-cultured cell proliferation and carotenoid accumulation (Zheng et al., 2020). The *Carotenoid Cleavage Dioxygenase 4b* gene contributes to picrocrocin biosynthesis and to the formation of volatile apocarotenoids. Despite remaining gaps in carotenoid profiling and callus formation in transgenic lines, overexpression of *CitCCD4b* demonstrates dual functionality in volatile compound synthesis (Zheng et al., 2021). β-Cyclocitral derivatives act as stress signaling molecules in response to salinity, drought, and high light, functioning as secondary messengers that regulate ¹O_2_-responsive gene expression through zinc-finger proteins. ¹O_2_, an electrophilic molecule with conjugated double bonds, participates in oxidative reactions that generate apocarotenoids such as β-cyclocitral (Ramel et al., 2012c).

β-Cyclocitral treatment also downregulated genes involved in chlorophyll biosynthesis, including protochlorophyllide reductase, copper target 1, *HEME1*, *PORB*, and *CHLM*, while upregulating the chlorophyll-degrading genes *CLH1* and *CLH2*. This coordinated regulation reduces chlorophyll content in vascular plants (Ramel et al., 2013b).

In addition to this enzymatic route, β-cyclocitral is also produced non-enzymatically through the oxidation of β-carotene by ¹O_2_ generated in PSII under high light, drought, salinity, or heat; in cyanobacteria, a dedicated β-carotene 7, 8(7′, 8′)-oxygenase that releases β-cyclocitral has been characterized (Jüttner and Höflacher, 1985). The volatile β-cyclocitral is subsequently oxidized to the more stable, water-soluble β-CCA, which accumulates strongly under drought and acts as a mobile stress signal (D'Alessandro et al., 2019; Lachica et al., 2026) (**Figure 1**).

## 3 β-Cyclocitral signalling and hormonal interactions

Before considering each hormone in turn, it is worth being clear about what is firmly established and what is still inferred. The best-characterised mode of β-cyclocitral signalling is an ABA-independent, chloroplast-to-nucleus retrograde pathway: β-cyclocitral, together with its stable derivative β-cyclocitric acid, triggers a SCARECROW-LIKE 14 (SCL14)–TGA (TGACG-motif-binding) class II xenobiotic-detoxification response and acts through the nuclear zinc-finger protein MBS1, while β-cyclocitric acid additionally engages the cell-cycle regulator SIAMESE-RELATED 5 (SMR5) and the cytochrome P450 CYP81D11 (D'Alessandro et al., 2018; Shumbe et al., 2017; Braat et al., 2023; Lachica et al., 2026). By contrast, the specific connections to the ABA, SA and JA signalling modules discussed below are, for the most part, inferred from the general biology of these hormones rather than demonstrated directly for β-cyclocitral. We therefore present them as a working model that points to plausible sites of convergence and to clear targets for future experiments.

### 3.1 β-cyclocitral and abscisic acid

β-Cyclocitral, a carotenoid-derived apocarotenoid, serves as a multifaceted signaling molecule that orchestrates plant responses to abiotic stresses, particularly drought and high light, by integrating hormonal, molecular, biochemical, and developmental pathways. At the molecular level, β-cyclocitral modulates the ABA signaling network, a central regulator of the drought response (Carnà et al., 2025). One possibility is that β-cyclocitral intersects with the core ABA module, in which sucrose non-fermenting 1-related protein kinases 2 (SnRK2s) act as positive regulators downstream of the PYR/PYL receptors and PP2C phosphatases (Iglesias-Moya et al., 2024; Wang et al., 2023). Activated SnRK2s phosphorylate downstream transcription factors, including ABF/AREB (ABA-responsive element binding factors) thereby promoting the transcription of ABA-responsive genes involved in stomatal regulation, osmolyte biosynthesis, antioxidant defense, and stress-protective protein accumulation. β-Cyclocitral-induced ROS could in principle act as secondary messengers that further stimulate SnRK2 activity and stress-responsive gene expression, forming a feedback loop between hormonal and redox signals; to our knowledge, however, this feedback has not yet been demonstrated directly for β-cyclocitral.

In tomato plants subjected to prolonged water deficit, β-cyclocitral-treated plants maintained high relative water content (RWC) and exhibited no leaf flaccidity or curling, which are typical drought symptoms (Deshpande et al., 2021a). Interestingly, this enhanced RWC occurred despite partially open stomata and reduced ABA concentrations, indicating that mechanisms other than ABA-dependent stomatal closure contribute to water conservation. One key mechanism involves root system architecture. β-Cyclocitral promotes meristematic cell division in root apices and lateral root emergence, as observed in both tomato and *Arabidopsis* seedlings, resulting in a more extensive and functionally efficient root network. Enhanced root proliferation increases soil water uptake, sustaining hydration during early and mature plant development (Zhang et al., 2022).

Biochemically, β-cyclocitral regulates osmotic homeostasis by inducing the accumulation of compatible solutes, particularly proline. Proline functions as an osmolyte to stabilize cellular turgor, maintain leaf water potential, and buffer intracellular osmotic stress. Additionally, proline acts as a ROS scavenger and a signaling molecule that activates antioxidant enzymes, including superoxide dismutase (SOD), catalase (CAT), and glutathione peroxidase (GPX), enhancing the plant’s oxidative-stress tolerance. β-Cyclocitral also promotes the uptake and redistribution of inorganic ions such as K^+^, Ca²^+^, and Mg²^+^, further contributing to osmotic adjustment and ion homeostasis under water-limiting conditions. These combined effects reduce dehydration-induced cellular damage and prevent leaf wilting, even when stomatal conductance remains relatively high. Importantly, osmolyte accumulation and ion homeostasis are themselves classical outputs of ABA signaling: although several of these responses can proceed independently of ABA (Deshpande et al., 2021a), they overlap with, and are amplified by, ABA-dependent transcription, because β-cyclocitral-enhanced SnRK2 activity promotes ABF/AREB-driven expression of osmolyte-biosynthesis, ion-transport, and antioxidant genes. Proline accumulation and ion homeostasis therefore represent shared endpoints of β-cyclocitral and ABA signaling rather than a purely ABA-independent branch. That said, on current evidence the drought protection conferred by β-cyclocitral is predominantly ABA-independent; in primed tomato it developed despite lowered ABA and only partially closed stomata (Deshpande et al., 2021a), and the best-characterised β-cyclocitral response, the SCL14–TGA II detoxification pathway, operates independently of ABA (D'Alessandro et al., 2018). The SnRK2–ABF/AREB link suggested above should therefore be read as a candidate point of convergence rather than an established requirement.

At the developmental level, the impact of β-cyclocitral extends beyond root architecture to overall plant growth and stress resilience. By integrating signaling between ABA, ROS, and carotenoid-derived apocarotenoids, β-cyclocitral primes plants for rapid adaptation to fluctuating water availability. Enhanced root proliferation increases soil-exploration capacity, while osmolyte accumulation and ion homeostasis maintain cellular hydration. Notably, this improved water status is achieved even though a large fraction of stomata remain partially open and stomatal conductance stays relatively high, so that CO_2_ assimilation and photosynthetic efficiency are preserved under drought (Deshpande et al., 2021a). How plants treated with β-cyclocitral sustain their water status despite this high conductance is not yet resolved, and is considered further in Section 4.3.

### 3.2 β-cyclocitral and salicylic acid

β-Cyclocitral, a chloroplast-derived apocarotenoid formed through the oxidative cleavage of β-carotene under high light intensity, drought, salinity, or other stress-induced ROS surges, has emerged as a central retrograde signaling molecule with profound crosstalk with SA at multiple regulatory levels, shaping the plant’s adaptive and defense responses (Li and Kim, 2021). Physiologically, β-cyclocitral functions as a rapid stress signal capable of enhancing thermotolerance, stabilizing PSII reaction centers, improving electron transport, and reducing ¹O_2_ accumulation by activating photoprotective mechanisms such as non-photochemical quenching (NPQ) and the xanthophyll cycle; SA complements these effects by improving photosynthetic resilience through modulation of stomatal conductance, maintenance of Rubisco activity, enhanced expression of chlorophyll-binding proteins, and strengthening of antioxidant capacity. Together, β-cyclocitral and SA form a synergistic regulatory system that fine-tunes metabolic and physiological homeostasis under abiotic stress.

At the molecular level, β-cyclocitral may also intersect with SA signalling. One proposed interaction involves the redox-dependent activation of NPR1 (Nonexpressor of Pathogenesis-Related Genes 1), the master transcriptional coactivator of the SA pathway. Under unstressed conditions, NPR1 remains in the cytosol as an oligomer linked by disulfide bonds; upon SA accumulation, a shift in cellular reduction potential together with thioredoxin-catalysed reduction of these disulfides releases NPR1 monomers, which then translocate into the nucleus (Mou et al., 2003; Tada et al., 2008). Because β-cyclocitral perturbs the cellular thiol redox state, it could in principle modulate this step, but a direct effect of β-cyclocitral on NPR1 monomerization has not been demonstrated. Once inside the nucleus, monomeric NPR1 interacts with TGA transcription factors to activate the transcription of SA-responsive defense genes, including *PR1*, *PR2*, *PR5*, and other pathogenesis-related (PR) proteins involved in ROS detoxification, redox regulation, osmoprotection, and cellular repair. Furthermore, β-cyclocitral has been suggested to activate mitogen-activated protein kinase (MAPK) cascades (e.g., *MPK3*, *MPK6*), which could phosphorylate SA-regulated transcription factors, thereby enhancing their DNA-binding activity and accelerating transcriptional reprogramming under stress. The interplay between β-cyclocitral and SA also extends to chromatin remodeling, where β-cyclocitral-regulated ROS bursts influence histone acetylation and methylation patterns that facilitate enhanced transcription of SA-dependent defense genes during stress priming (Xing et al., 2013).

At the systems level, we envisage that β-cyclocitral–SA crosstalk forms an integrative signaling network linking chloroplast retrograde communication, redox homeostasis, transcriptional regulation, and defense-gene activation. β-Cyclocitral acts upstream by sensing stress-triggered chloroplast oxidation and transmitting this information to the nucleus to activate SA biosynthetic machinery, notably the isochorismate route to SA (Wildermuth et al., 2001), and the associated signalling components. SA, in turn, strengthens antioxidant defense and photoprotective pathways, supporting β-cyclocitral functions and ensuring more stable chloroplast performance under continuous stress. This reciprocal reinforcement creates a positive feedback loop in which β-cyclocitral enhances SA accumulation and signaling, and SA sustains cellular conditions that maintain β-cyclocitral-dependent stress acclimation. Together, these molecules orchestrate a coordinated defense system that enables plants to detect stress earlier, activate transcriptional responses faster, maintain redox equilibrium, and achieve more durable tolerance to diverse abiotic stresses, demonstrating that β-cyclocitral functions not only as a chloroplast oxidative signal but also as a central amplifier and modulator of SA-governed molecular defense networks (García et al., 2010).

### 3.3 β-cyclocitral and jasmonic acid

β-Cyclocitral, a chloroplast-derived oxidative apocarotenoid generated by ROS-mediated cleavage of β-carotene, exhibits highly integrated and multilayered crosstalk with JA, creating a sophisticated oxidative–lipid signaling network essential for plant tolerance to abiotic stresses such as high light, drought, salinity, heat, and heavy metals (Felemban et al., 2019). JA itself is a well-established regulator of plant responses to these stresses (Ali and Baek, 2020). Their combined action forms a highly efficient physiological shield that strengthens redox buffering, chloroplast integrity, carbon balance, and global metabolic homeostasis.

Biochemically, β-cyclocitral amplifies stress-induced lipid remodeling by activating phospholipases (PLA1/PLA2), galactolipid lipases, and ROS-sensitive lipid-transfer proteins, increasing substrate availability for JA biosynthesis while stimulating the expression of lipoxygenase (LOX), allene oxide synthase (AOS), and allene oxide cyclase (AOC), which accelerate the formation of 12-oxo-phytodienoic acid (OPDA) (Song et al., 2025). β-Cyclocitral also promotes the synthesis of electrophilic oxylipins and reactive carbonyl species (RCS), which act as secondary messengers influencing JA biosynthesis and JA-dependent gene regulation. At the same time, JA supports β-cyclocitral generation by enhancing plastoglobuli remodeling, carotenoid turnover, and the ROS sensitivity of thylakoid membranes, creating a positive-feedback loop between the β-cyclocitral and JA pathways (Knieper et al., 2023).

At the molecular level, β-cyclocitral is proposed to influence transcriptional, post-transcriptional, and post-translational regulation within the JA signaling framework. β-Cyclocitral-induced ROS may enhance activation of MAPK cascades (*MPK3*/*MPK6*), which could phosphorylate *MYC2*, *MYC3*, and *MYC4*, the central JA-responsive transcription factors, thereby increasing their transcriptional activity and stability. β-Cyclocitral increases nuclear redox signaling by raising the GSH/GSSG and ASC/DHA ratios, modifying the redox state of thiol-containing proteins that regulate JA-responsive transcription factors and chromatin-modifying enzymes. β-Cyclocitral-driven redox waves facilitate histone acetylation and demethylation at JA-responsive promoters through redox-sensitive chromatin modifiers such as histone deacetylases (HDACs), SWI/SNF complexes, and histone acetyltransferases (HATs), creating a transcription-permissive chromatin landscape. JA, in turn, strengthens β-cyclocitral-triggered retrograde signaling via upregulation of genes associated with chloroplast proteostasis (FtsH and Clp proteases), ROS-scavenging systems (APX, GPX, PrxQ), and plastid–nucleus communication proteins (ANAC family). JA and β-cyclocitral further interact through *ALTERNATIVE OXIDASE* (*AOX*)-mediated mitochondrial retrograde signaling, which integrates ROS pulses from chloroplasts with JA-driven defense transcription.

**Figure 2:**
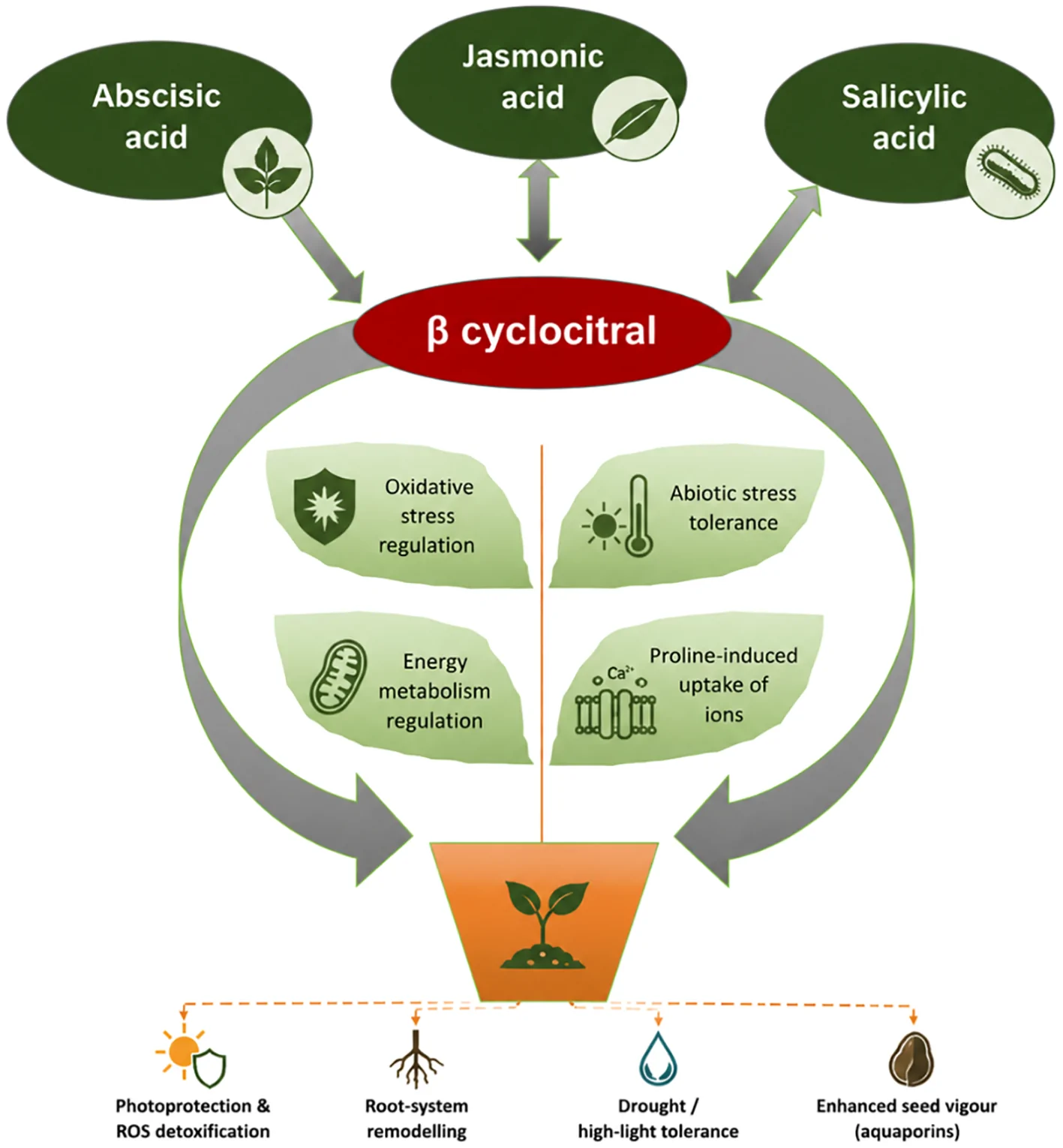
Schematic representation of crosstalk between β-cyclocitral and abscisic acid, jasmonic acid, and salicylic acid.

**Figure 3:**
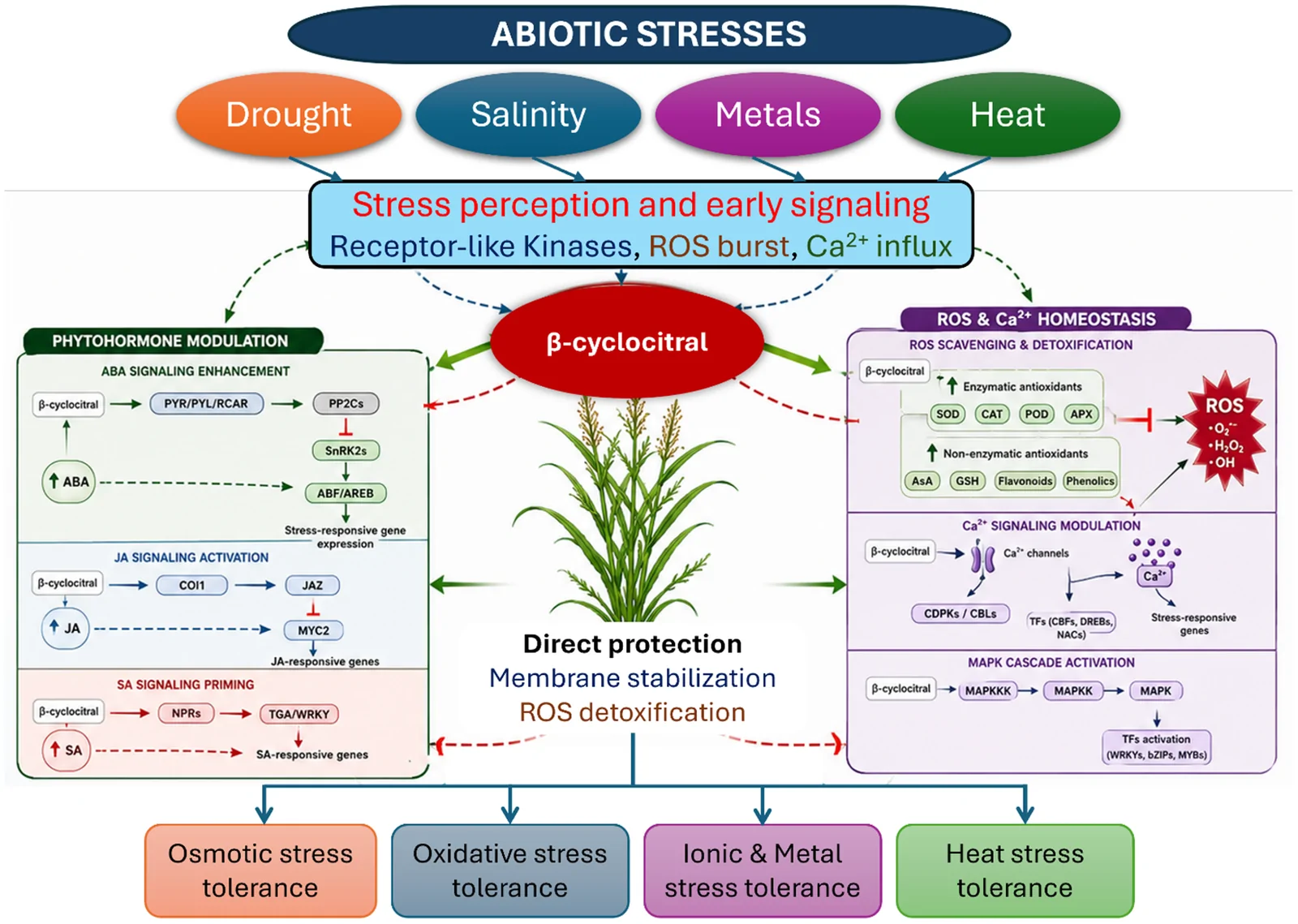
Mechanistic role of β-cyclocitral in abiotic stress tolerance through phytohormonal crosstalk and signaling reprogramming. Stress perception (receptor-like kinases, ROS burst, Ca²^+^ influx) activates β-cyclocitral, which engages three modules: phytohormone signalling (SnRK2–ABF/AREB, COI1–JAZ–MYC2, NPR1–TGA/WRKY), ROS/Ca²^+^ homeostasis with MAPK cascades, and direct membrane/ROS protection, conferring osmotic, oxidative, ionic, and heat-stress tolerance.

The three hormone pathways described above do not act in isolation but converge on β-cyclocitral, which integrates them into a coordinated regulatory network. **Figure 2** illustrates this central role, in which β-cyclocitral interacts with the three major phytohormones (ABA, JA, and SA) to modulate plant adaptation under adverse environmental conditions, while the underlying molecular architecture of the crosstalk, comprising the receptor-level hormone modules (SnRK2–ABF/AREB, COI1–JAZ–MYC2, and NPR1–TGA/WRKY), ROS and Ca²^+^ homeostasis, and MAPK cascades, is detailed in **Figure 3**.

### 3.4 Omics perspectives on β-cyclocitral–hormone crosstalk

Multi-omics approaches have become central to placing β-cyclocitral within the broader hormone-signalling network and to distinguishing antagonistic from synergistic responses under combined stresses. The integration of transcriptomics, proteomics, metabolomics, and epigenomics now provides the standard framework for dissecting how β-cyclocitral and the defense hormones jointly reprogram plant metabolism, since each layer constrains and complements the others (Roychowdhury et al., 2023; Varadharajan et al., 2025).

Large-scale transcriptome profiling after SA or JA treatments, often using mutants, provides genome-wide maps of antagonistic and synergistic gene sets, while ChIP-seq of MYC and WRKY factors reveals overlapping or mutually exclusive promoter-occupancy patterns that explain transcript-level antagonism. Proteomics and phosphoproteomics document modifications of transcription factors that alter their activity and interactions in the presence of the other hormone (**Table 1**). Metabolomic and oxylipin profiling reveals that SA treatment can reduce JA and OPDA accumulation, and vice versa, in some contexts, whereas in others both metabolites increase; time-series omics are especially informative, since immediate-early genes show one type of interaction while late genes can be synergistic as networks rewire (Rachowka et al., 2025).

**Table 1:** Key genes and pathways implicated in crosstalk between β-cyclocitral, ABA, SA, and JA.

Gene/component	Hormones involved	Functional role	Evidence supporting crosstalk	References
*CCD4*/*CCD1*	β-cyclocitral, ABA	Carotenoid cleavage precursor synthesis	Shared carotenoid-derived metabolic pathway	Ramel et al., 2012c; Lachica et al., 2026
*TGA2/5/6*	β-cyclocitral, SA	SA-responsive TFs; partners of *SCL14*	Shared TGA-mediated signaling regulation	Fode et al., 2008; Carnà et al., 2025
*NPR1*	SA, JA	Master regulator of SA; suppresses JA branch	Redox-mediated NPR1 signaling crosstalk	Spoel and Dong, 2008; Duhan and Pasrija, 2025
*DREB2A*/*DREB2B*	ABA, β-cyclocitral	Drought and heat stress TFs	Enhanced antioxidant-mediated DREB activation	Chen et al., 2022; Varadharajan et al., 2025
*WRKY33*/*WRKY40*/*WRKY70*	SA, JA, ABA	Hormonal-crosstalk transcriptional integrators	WRKY-mediated SA–JA crosstalk regulation	Ülker et al., 2007; Duhan and Pasrija, 2025
*MPK3*/*MPK4*/*MPK6*	β-cyclocitral, ABA, SA, JA	Stress-activated MAPKs	ROS–MAPK-mediated hormonal crosstalk	Ramel et al., 2013b; Duhan and Pasrija, 2025
*AOX1a*	β-cyclocitral, ABA	Retrograde signaling modulator	AOX1a-mediated stress signaling	Giraud et al., 2009; Lachica et al., 2026
*SCL14*	β-cyclocitral, SA	Activates the TGA II detoxification (SORG) response	β-cyclocitral-induced SCL14–TGA II module drives detoxification genes	D'Alessandro and Havaux, 2019; Carnà et al., 2025
*MBS1*	β-cyclocitral	Mediator of singlet-oxygen retrograde signaling	β-cyclocitral up-regulates MBS1 for photoacclimation	Shumbe et al., 2017; Lachica et al., 2026
*CYP81D11*/*GSTU*	β-cyclocitral	Detoxification and antioxidant SORG effectors	β-cyclocitral-induced detoxification of reactive electrophiles	Shumbe et al., 2017; Carnà et al., 2025
*EXECUTER1/2* (*EX1*/*EX2*)	β-cyclocitral	Chloroplast singlet-oxygen sensing	Parallel singlet-oxygen sensing converging with β-cyclocitral signaling	Dogra et al., 2018; Zhang et al., 2023
*PYR*/*PYL*–*PP2C*–*SnRK2*	ABA	Core ABA perception and signal transduction	β-cyclocitral and ABA converge on stomatal and drought responses	Nambara and Marion-Poll, 2005; Iglesias-Moya et al., 2024
*MYC2*–*JAZ*/*COI1*	JA	Master JA receptor–repressor–TF module	SA/NPR1 antagonism of MYC2 integrates with β-cyclocitral–ROS cues	Luo et al., 2023

Comparative transcriptomes of β-cyclocitral-treated plants are especially informative; in tomato, exogenous β-cyclocitral reconfigured the expression of stress-responsive genes, with most up-regulated transcripts mapping to abiotic- and biotic-stress categories (Deshpande et al., 2021b), whereas ¹O_2_-driven transcriptome reprogramming reshaped cell-wall and stomatal-development programs, consistent with β-cyclocitral acting downstream of ¹O_2_ (Zhang et al., 2023). Time-resolved profiling further links this retrograde input to hormonal balance, implicating regulators such as ANNEXIN1 in coordinating light sensitivity with SA-, JA-, and ABA-dependent outputs (Rachowka et al., 2025).

Paired transcriptome–metabolome studies are increasingly resolving the biochemical endpoints of this crosstalk, showing how shifts in oxylipin and defense-metabolite pools accompany the transcriptional antagonism captured by mutant- and ChIP-based analyses (Han et al., 2023; Duhan and Pasrija, 2025). Nevertheless, most current datasets remain correlative and tissue-averaged, and very few simultaneously profile β-cyclocitral or β-cyclocitric acid alongside hormone metabolites. Emerging single-cell and spatial omics, quantitative phosphoproteomics of SnRK2-, MAPK-, and NPR1-centered signaling modules, and machine-learning-assisted multi-omics integration therefore represent priority directions for converting the descriptive maps summarized in **Table 1** into causal, cell-type-resolved models of β-cyclocitral–hormone signaling (Varadharajan et al., 2025).

## 4 Role of β-cyclocitral in plant defence against abiotic stress

### 4.1 β-cyclocitral, reactive oxygen species, and the stress-signalling axis

The link between abiotic stress and β-cyclocitral is mechanistic and sequential. Drought, salinity, heat, and excess light all restrict CO_2_ fixation and over-reduce the photosynthetic electron transport chain, which promotes the formation of ¹O_2_ and other ROS in PSII. Because β-carotene is embedded in the PSII reaction center, this ¹O_2_ oxidizes β-carotene and releases β-cyclocitral in proportion to the severity of the stress. β-Cyclocitral, together with its stable derivative β-CCA, then acts as a diffusible retrograde signal that travels from the chloroplast to the nucleus and reprograms the expression of detoxification and stress-acclimation genes (the SORGs). Thus, the ROS burst that accompanies stress is not only a source of damage but also the trigger that generates the β-cyclocitral signal, and it is this signaling function, rather than ROS accumulation per se, that underlies β-cyclocitral-mediated tolerance. The evidence for each step of this pathway is summarized below.

**Figure 4:**
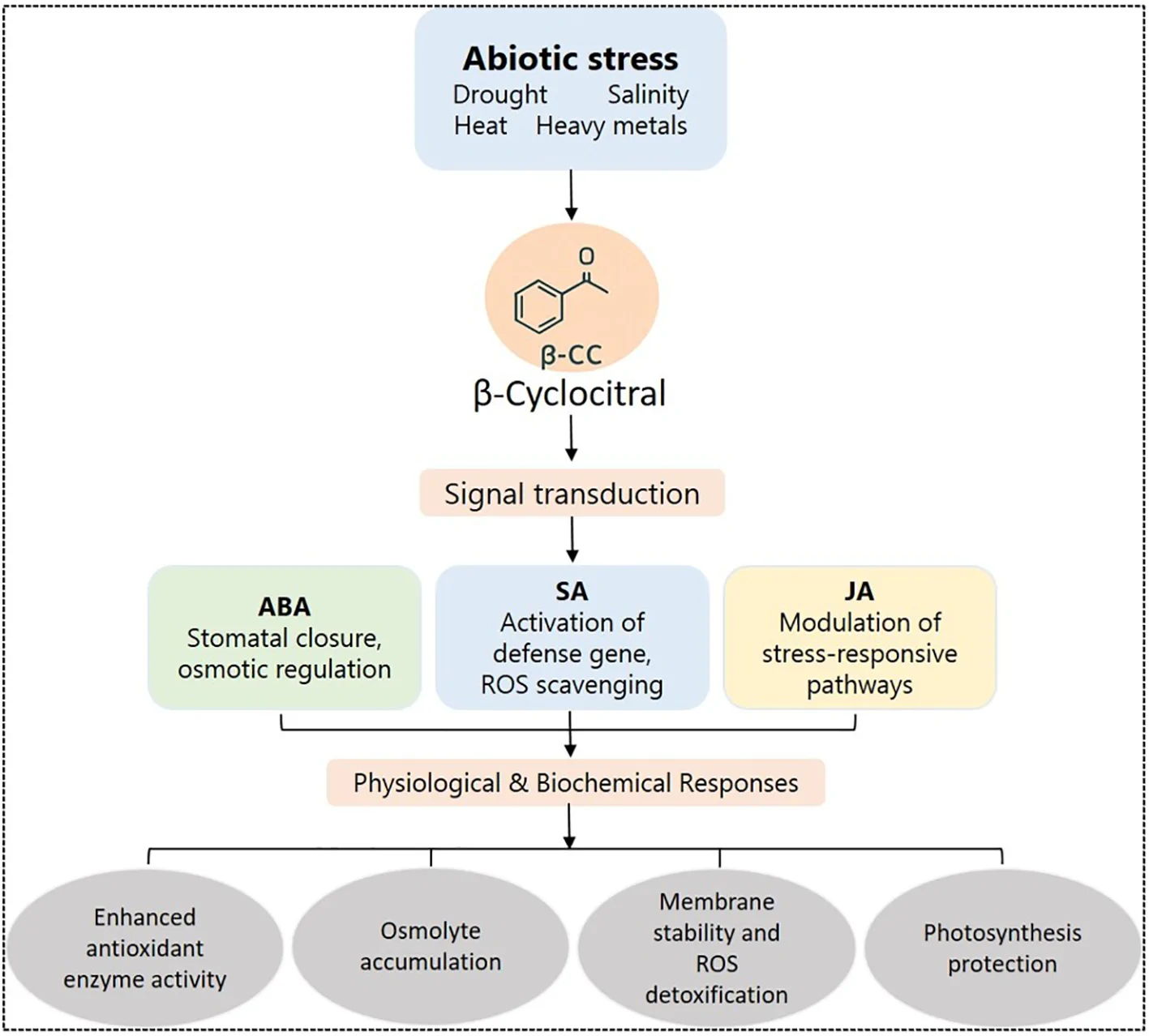
Schematic representation of β-cyclocitral-mediated enhancement of plant tolerance under abiotic stress such as drought, salinity, heat, and heavy metals. β-CC, β-cyclocitral.

β-Cyclocitral is a novel plant signal that promotes stress tolerance in both vascular and non-vascular plants, and it is crucial for drought-stress tolerance. Photosynthesis, stomatal conductance, and the respiration and signal-transduction systems are all negatively affected by drought stress (**Figure 4**). Under drought stress, plant production, cell growth, and leaf senescence are all dramatically affected (Misra et al., 2020). According to Tanveer et al. (2019), ROS generated by drought stress threaten oxidative balance, carbon assimilation, and antioxidant systems. The relationship between β-cyclocitral and ROS during drought is illustrated by the observation that increasing β-cyclocitral levels help reduce the β-carotene content in *Solanum lycopersicum*. Overexpression of SOD and CAT increased their transcript levels in plants under drought stress, enhancing stress resistance. The generation of ¹O_2_, which acts as a signaling molecule in stressed plants, drives lipid peroxidation. High levels of SORGs in *Arabidopsis thaliana* are a key sign of impending stress. Compared with plants grown under control or naturally stressful conditions, application of β-cyclocitral reduces photoinhibition. β-Cyclocitral functions as a secondary messenger that is relayed to the nucleus via the MBS1 protein (Shumbe et al., 2017). ROS are produced when chlorophyll becomes overexcited.

According to Isayenkov (2012), salt stress impairs plant growth, ionic balance, nutrient homeostasis, and stomatal function in shoots. Under salinity stress, plants experience impaired water uptake, reduced water potential, oxidative stress, and programmed cell death (PCD). When ROS levels rise, ¹O_2_ is generated in PSII complexes through energy transfer from triplet chlorophyll (³Chl*). Ramel et al. (2012c) reported that β-cyclocitral acts as a stress signal and a likely messenger in the ¹O_2_ defense and signaling pathway in plants. They observed changes in the expression of ¹O_2_-responsive genes in *Arabidopsis* following β-cyclocitral treatment and found that it enhanced tolerance to photooxidative stress through reprogramming of gene expression. Apocarotenoids other than β-cyclocitral also help reduce photooxidative stress by inducing gene-expression patterns associated with high tolerance; one example is dihydroactinidiolide, produced by the ¹O_2_-mediated oxidation of the carotene-derived β-ionone. Plants pretreated with volatile β-cyclocitral showed reduced lipid peroxidation and PSII photoinhibition under high-light stress compared with untreated *Arabidopsis* plants (Shumbe et al., 2017).

**Figure 5:**
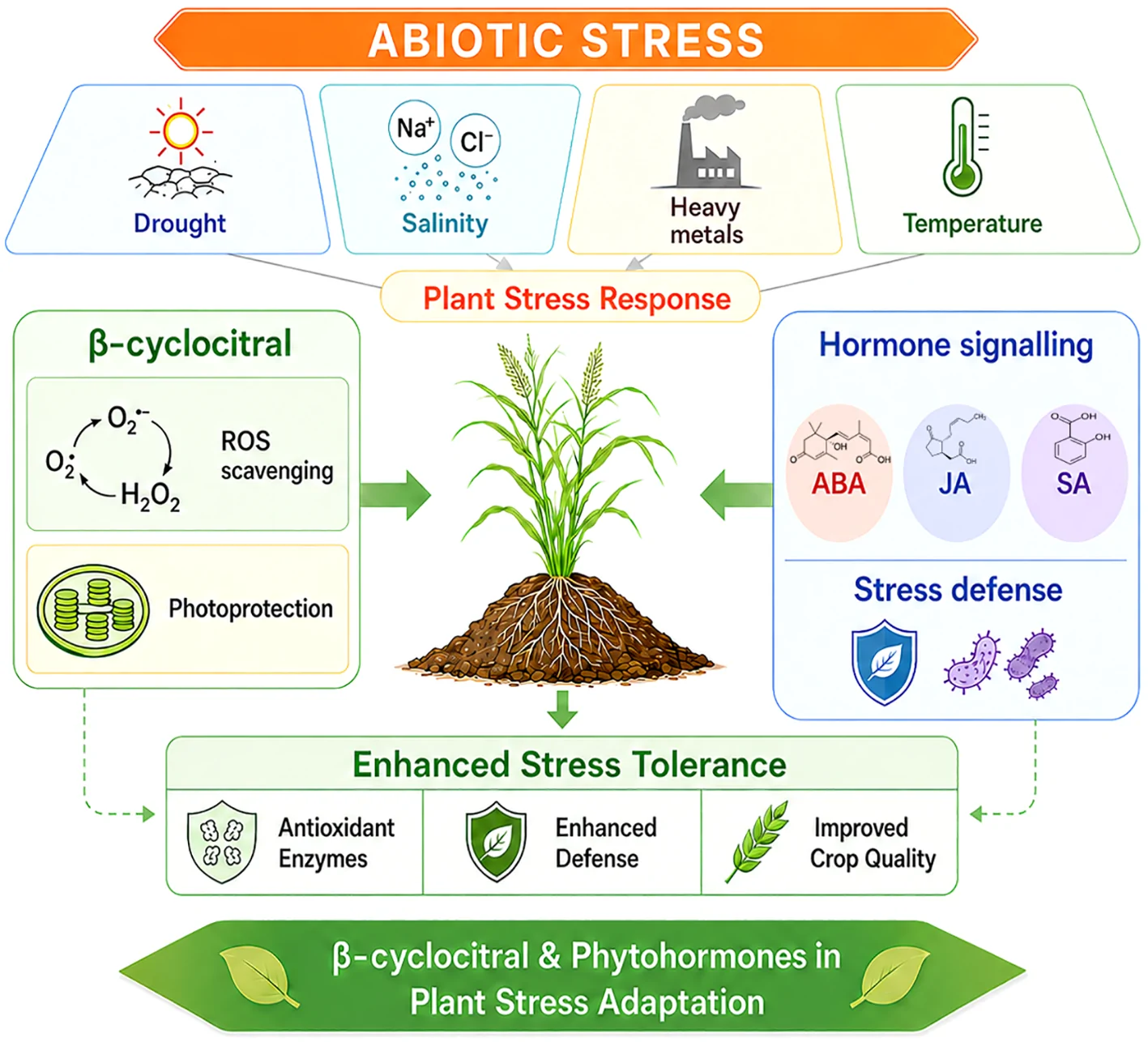
Schematic representation of β-cyclocitral- and phytohormone-mediated regulation of plant stress responses under abiotic stresses. Green arrows indicate the parallel contributions of β-cyclocitral and of hormone signalling to stress tolerance.

Acting together, β-cyclocitral and the stress hormones translate this signalling into concrete protective outcomes: enhanced antioxidant-enzyme activity, membrane stabilization, and improved crop performance, as summarized in **Figure 5**.

### 4.2 Photosynthesis and photoprotection

A higher photosynthetic rate with enhanced chlorophyll content is seen in β-cyclocitral-treated, drought-exposed plants. Drought-exposed plants treated with β-cyclocitral show enhanced root and shoot growth, indicating improved tolerance to drought stress (Ferreira Júnior et al., 2018). Drought stress often leads to growth inhibition and a decline in photosynthetic capacity. In drought-sensitive plants, reduced chlorophyll content makes them particularly prone to photo-oxidation. Notably, β-cyclocitral treatment has been shown to double the photosynthetic rate, which in turn promotes enhanced root development and increased stalk length. These findings provide strong evidence that β-cyclocitral can improve plant resilience and survival under water-deficit conditions. The exchange of oxygen and CO_2_ supports respiration and photosynthesis, both regulated by ABA. Photo-oxidative damage is mitigated by protecting the photosynthetic apparatus through the consumption of ¹O_2_ and the quenching of triplet chlorophyll. A reduction in CO_2_ influx results from limited stomatal conductance, which in turn lowers the photosynthetic rate and growth under stress conditions. Experimental data have shown that enzymes and photosynthetic activators help increase the photosynthetic rate and carbon accumulation (Schwachtje et al., 2006).

### 4.3 Stomatal regulation

To characterize the stomatal response, the proportions of fully open, partially open, and closed stomata were scored. In β-cyclocitral-treated plants under drought, a larger fraction of stomata remained partially open, so that stomatal conductance stayed high even though bulk ABA levels were unchanged. Stomatal conductance was twice as high in β-cyclocitral-treated plants as in water-treated plants. Under water stress, ABA, itself an apocarotenoid, mediates stomatal closure, thereby lowering transpiration and water loss (Nguyen et al., 2016). Stomatal density and stomatal index were also examined before and after β-CCA treatment, since a small reduction in these parameters can enhance water-use efficiency (Yongchao et al., 2023). The greater number of partially opened stomata presumably permits CO_2_ inflow, which raises the rate of photosynthetic activity. Although ABA signaling primarily controls stomatal closure, other regulators that show functional convergence with ABA have also been identified as drought-induced mediators of stomatal closure. The jasmonic acid precursor 12-oxo-phytodienoic acid (12-OPDA) also promotes stomatal closure (Savchenko et al., 2019). Thus, transpirational water loss is reduced and RWC is elevated. It therefore remains unclear how β-cyclocitral-treated plants maintain RWC despite their higher stomatal conductance. It was recently demonstrated that, in tomato seedlings, β-cyclocitral promotes cell proliferation in the root meristem, which in turn increases root development and branching. However, stomatal closure is not a prerequisite for the protective effects of β-cyclocitric acid (D'Alessandro and Havaux, 2019).

### 4.4 Root-growth promotion

β-Cyclocitral plays a significant biological role in regulating root growth. Plants treated with β-cyclocitral exhibit reduced visible wilting under drought stress, highlighting its protective role in improving tolerance to water deficit. Both drought-stressed and well-watered plants supplemented with β-cyclocitral show increased accumulation of proline and SOD, indicating enhanced osmotic adjustment and antioxidant defense. Moreover, β-cyclocitral improves carbon allocation within the plant, thereby facilitating water uptake and promoting the development of elongated root systems.

These stimulatory effects are particularly evident during the seedling stage, where the enhancement of root growth is more pronounced than in mature plants. By improving water absorption and maintaining leaf water balance, β-cyclocitral contributes to higher RWC, thereby supporting physiological stability under stress conditions. Similar findings were reported by Deshpande et al. (2021a), who demonstrated in tomato seedlings that β-cyclocitral treatment stimulated meristematic cell division in roots, resulting in enhanced root formation and increased lateral root branching. Reduced endogenous apocarotenoid levels increased root sensitivity, whereas inhibition of CCDs reduced lateral root proliferation by approximately 50%; this inhibitory effect was subsequently reversed through exogenous β-cyclocitral application.

Comparable observations have also been reported in *Arabidopsis*, where β-cyclocitral significantly promoted root growth and development (D'Alessandro and Havaux, 2019; Dickinson et al., 2019). It should be noted that the oxidised derivative β-cyclocitric acid has the opposite effect on root growth, which it inhibits through induction of the cell-cycle inhibitor *SMR5* (Braat et al., 2023; see Section 5.1). Interestingly, the β-cyclocitral-induced enhancement in photosynthetic activity preferentially stimulates root growth rather than shoot growth, indicating a strategic allocation of assimilates toward belowground structures. Furthermore, β-cyclocitral strengthens root architecture by increasing root sturdiness and robustness. In rice, β-cyclocitral application alleviates the detrimental effects of salinity on rooting depth, enabling plants to maintain stronger root systems and improved vigor under salt-stress conditions.

## 5 Roles of β-cyclocitral in nodulation, ripening, and senescence

### 5.1 Nodulation and root organogenesis

β-Cyclocitral has also been reported to enhance nodule formation in plants (Carnà et al., 2025). Carotenoid-derived apocarotenoids are increasingly recognized as regulators of root–microbe symbioses. Strigolactones, which are themselves produced by carotenoid cleavage, act as rhizosphere signals that promote arbuscular mycorrhizal colonization and modulate nodulation, and additional apocarotenoids accumulate during these interactions. Within this context, β-cyclocitral has been reported to enhance nodule formation, consistent with its broader role as a regulator of root growth and root architecture; however, the precise molecular mechanism by which β-cyclocitral influences nodulation remains to be established and warrants dedicated study.

Because these effects parallel the auxin-independent promotion of lateral-root and meristem activity by β-cyclocitral, and because carotenoid-cleavage products such as strigolactones are established regulators of nodulation and mycorrhization, a central open question is whether β-cyclocitral acts through, or in parallel with, the strigolactone and Nod-factor signalling pathways during nodule ontogeny. Resolving this, for example by combining apocarotenoid quantification with nodulation phenotyping in CCD- and symbiosis-pathway mutants, is a priority that has not been addressed by earlier apocarotenoid reviews and represents a promising direction for improving symbiotic nitrogen fixation.

β-Cyclocitral is also an intrinsic regulator of root organogenesis. A chemical-genetic screen identified β-cyclocitral as an endogenous, conserved root-growth regulator that promotes stem-cell and meristematic divisions and stimulates lateral-root branching in *Arabidopsis*, tomato and rice (Dickinson et al., 2019). Notably, this activity was found to be independent of the auxin, brassinosteroid and reactive-oxygen-species pathways that classically drive meristem growth, which sets β-cyclocitral apart as a distinct carotenoid-derived cue that shapes root architecture during development. Importantly, the aldehyde and its oxidised derivative are not interchangeable in this respect: whereas β-cyclocitral stimulates root growth, β-cyclocitric acid inhibits root cell division and elongation by selectively inducing the cell-cycle inhibitor *SMR5* in root tip cells, thereby reorienting root metabolism from growth towards detoxification and suberin deposition and conferring drought tolerance (Braat et al., 2023). This contrast suggests that the balance between β-cyclocitral and β-cyclocitric acid, rather than either compound alone, shapes the root response to stress. Because a well-branched, deep root system underlies both efficient water and nutrient capture and effective nodule formation, the developmental control of root organogenesis by β-cyclocitral provides a mechanistic link between its growth-regulatory and symbiotic roles.

### 5.2 Senescence and ripening

Different growth regulators are involved in controlling physiological mechanisms in plant cells, tissues, organ development, dormancy regulation, flowering, and especially senescence (Sharma and Bons, 2025). During this complex biological process, the expression of genes associated with leaf senescence, macromolecule breakdown, and organelle degradation is modified. Leaf senescence, in turn, also affects plant stress-tolerance mechanisms. In addition, the ripening process is regulated by proteomic interactions, ripening-related receptor genes, and transcription factors across different crops. An increase in ABA levels promotes ROS generation, which induces oxidative stress and thereby causes cell damage and leaf senescence (Kim et al., 2021). Predominantly, ripening and senescence are governed by ethylene. ABA biosynthesis in the cytosol proceeds through the carotenoid pathway. Drought stress caused a considerable decline in plant yield and cell growth and accelerated leaf senescence. Evidence indicates that CCDs, together with regulatory proteins, influence diverse aspects of plant morphology, senescence, and plastid formation. ABA acts in concert with rice WRKY transcription factors, which accelerate leaf senescence by regulating biosynthetic genes and raising endogenous ABA levels (Kim et al., 2021).

β-Cyclocitral is directly implicated in these processes. Because progressive chlorophyll degradation is a hallmark of senescence, β-cyclocitral treatment reinforces this program by down-regulating chlorophyll-biosynthesis genes (e.g., *PORB*, *CHLM*, *HEME1*) while up-regulating the chlorophyllases *CLH1* and *CLH2*, thereby lowering chlorophyll content (Ramel et al., 2013b). During fruit ripening, extensive carotenoid remodeling and apocarotenoid turnover occur, and β-cyclocitral is among the volatile apocarotenoids released as β-carotene is metabolized; changes in its abundance therefore accompany the ripening transition and contribute to aroma (Felemban et al., 2019; Carnà et al., 2025). Because β-cyclocitral also modulates ABA- and ROS-dependent signaling, and because ABA and ethylene are the principal hormonal drivers of ripening and senescence, β-cyclocitral is well positioned to fine-tune the onset and progression of senescence in coordination with these hormones.

Whereas earlier apocarotenoid reviews have catalogued these functions only broadly, the specific contribution of β-cyclocitral as a ripening-associated volatile, released during climacteric carotenoid turnover and potentially feeding back on ABA and ethylene signalling to modulate the timing of ripening and leaf senescence, remains poorly resolved. Clarifying this feedback, and whether exogenous β-cyclocitral or β-CCA can be used to delay senescence or improve post-harvest quality, is an important and largely unexplored avenue for horticultural crop improvement.

**Figure 6:**
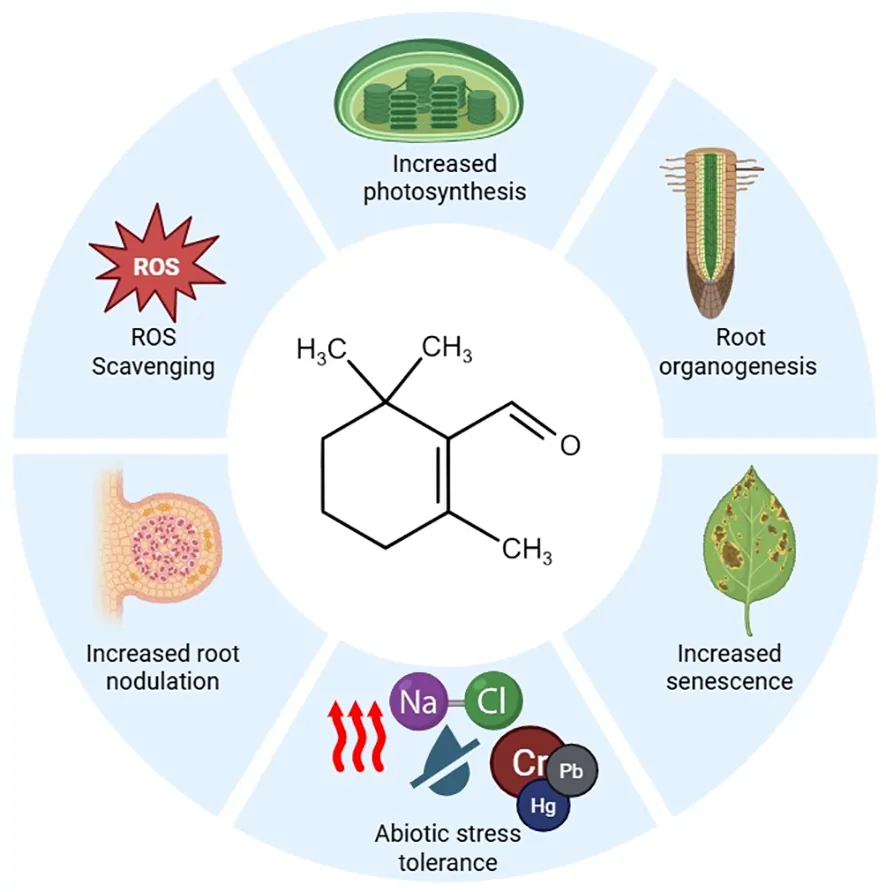
Role of β-cyclocitral in plant growth and development.

An integrated overview of these and the other physiological and stress-related roles of β-cyclocitral discussed throughout this review is provided in **Figure 6**.

## 6 Conclusion and prospects

β-Cyclocitral, a carotenoid-derived apocarotenoid, has emerged as a vital signaling molecule that enhances plant resilience to multiple abiotic stresses. β-Cyclocitral regulates root architecture, promotes nodule formation, improves photosynthesis and stomatal conductance, and modulates senescence and ripening, largely through its crosstalk with key phytohormones such as ABA, SA, and JA. Mechanistically, β-cyclocitral functions as a secondary messenger that mediates ROS signaling, triggers stress-responsive gene expression, and activates antioxidant defense systems, thereby mitigating photo-oxidative damage and maintaining water and nutrient homeostasis. The integrative role of β-cyclocitral in modulating physiological, molecular, and hormonal networks highlights its potential as a tool for enhancing crop stress tolerance. Future research focusing on the precise regulatory mechanisms of β-cyclocitral biosynthesis and signaling, and on its interactions with other phytohormones, will be critical for translating these findings into practical applications.
